# Mindfulness and psychological health in practitioners of Japanese martial arts: a cross-sectional study

**DOI:** 10.1186/s13102-020-00225-5

**Published:** 2020-12-07

**Authors:** Hiromitsu Miyata, Daisuke Kobayashi, Akifumi Sonoda, Hibiki Motoike, Saki Akatsuka

**Affiliations:** Faculty of Letters, Arts and Sciences, Waseda University, 1-24-1 Toyama, Shinjuku-ku, Tokyo, 162-8644 Japan

**Keywords:** Martial arts, Dispositional mindfulness, Subjective well-being, Depression, Long-term practice, Japanese

## Abstract

**Background:**

Empirical data have suggested that mind-body practices that originated in Eastern traditions can cause desirable changes to psychological traits, the brain, somatic physiological functions, etc. Martial arts in Japan refer to the physical/mental practices that were developed based on historical combat techniques. Today, martial arts are considered activities that seek embodiment and/or mind-body unity, as well as sports. Empirical studies involving practitioners of Japanese martial arts to date remain scarce.

**Methods:**

We conducted a questionnaire survey using a cross-sectional design to examine whether the practice of martial arts based on Japanese traditions are associated with mindfulness and psychological health. Participants included a population of practitioners of martial arts with a practice period of 0.6–35.0 years, and non-practitioners matched for demographic variables.

**Results:**

Compared with the non-practitioners, the practitioners of martial arts had significantly higher scores for mindfulness and subjective well-being and lower scores for depression. Among the practitioners of martial arts, a longer period of practice or a higher frequency of daily practice significantly predicted higher mindfulness and psychological health.

**Conclusions:**

The results obtained are consistent with those previously obtained for other populations of Japanese contemplatives, and support the view that practice of multiple Eastern mind-body practices might be associated with similar desirable psychological outcomes. A cross-sectional design has limitations in that it is difficult to determine the effect of continued practice, so that a longitudinal study that follows the same practitioners over time is desired in the future enquiry.

## Background

Increasing amounts of empirical data during recent decades have demonstrated that mind-body practices that originated in Eastern traditions including Buddhist meditation can cause desirable changes to psychological traits, the brain, somatic physiological functions, etc. (for reviews see [1, 2]). As Yasuo Yuasa noted, Eastern mind-body theory have not only considered body and mind as one, but also sought for mind-body integration and unity through physical and mental practices [3]. In Japan, such mind-body unity has traditionally referred to as “Shin-Shin Ichinyo (body and mind as one).” In addition, mindfulness has its origins in the Early Buddhism, and today refers to paying attention to one’s moment-to-moment awareness in nonjudgmental and nonreactive ways. The idea of mindfulness has proven effective in elucidating the psychological, physiological, and neurocognitive correlates of mind-body and/or contemplative practices in scientific and clinical contexts. Interventions based on mindfulness have been applied to various clinical and educational settings (for reviews see [4, 5]). For example, mindfulness-based stress reduction (MBSR) program developed by Jon Kabat-Zinn has been shown to reduce psychological symptoms in patients with chronic pain [6, 7]. Mindfulness-based cognitive therapy (MBCT) program has applied mindfulness to prevent the relapse of depression [8]. .b (dot-be) program is a mindfulness program developed for school children [9] and have proven effective for enhancing psychological health outcomes such as reduced stress and anxiety and increased psychological well-being.

Despite this accumulated evidence, in Japan, relatively little empirical data exist regarding practice-induced changes in the psychological status and/or somatic physiological states of practitioners of traditional mind-body and/or contemplative practices. Miyata et al. [10] included practitioners of traditional yoga in Japan whose practice period was 0.3 to 34.0 years and found that the practitioners self-reported higher mindfulness and subjective well-being and lower depression and negative affect compared with non-practitioners. In addition, a longer period and a larger amount of daily yoga/meditation practice were significantly associated with higher mindfulness and psychological health (see also [11]). Miyata and Sasaki [12] studied trainees of the Park-Sasaki method of speed-reading, a meditation-based technique in Japan to cultivate efficient reading strategies, and found that the trainees exhibited not only higher reading speeds but also higher mindfulness and psychological health than untrained participants. Among these trainees, a longer period of training and a higher reading speed were associated with higher mindfulness and psychological health (see also [13–15]). Given the consistency of results obtained from multiple populations, an important challenge is to examine the extent to which comparable psychological outcomes are observed for practitioners of various Japanese mind-body practices.

Martial arts in Japan refer to a variety of physical and mental practices that have been developed based on historical combat techniques. Today, martial arts are considered not only sports and/or fighting methods, but also activities that seek to obtain practical understanding of embodiment, mind-body unity, and/or physical and mental health [16]. These characteristics of marital arts are considered to converge with the nature of Zen Buddhism as well as that of mindfulness, and are referred to by the phrase “Ken-Zen Ichinyo (oneness of Zen and swordsmanship)” [17]. For example, karate (empty hand) is a traditional form of martial arts originated in Okinawa, Japan. Traditional karate involves not only body manipulation to efficiently defeat one’s opponent with bare hands, but also cultivating awareness to one’s breath and body through practice, as with Zen and mindfulness. Karate was later introduced to the mainland of Japan by experts such as Gichin Funakoshi, where elements of sports such as games has been largely incorporated [16]. Based on these historical backgrounds, it seems reasonable to assume that martial arts have similar correlates to mindfulness as well as sports in the context of psychological studies [16].

Empirical studies in recent years have started to demonstrate the effectiveness of interventions based on martial arts in the clinical context. For example, Tai Chi is a Chinese martial art that involves multiple components such as musculoskeletal training and enhancement of both interoceptive and exteroceptive awareness [18, 19]. Burschka et al. [19] studied multiple sclerosis patients and showed that a 6-month Tai Chi intervention was effective in reducing depression and increasing life satisfaction, as well as improving body balance and coordination. Tai Chi interventions have also proven effective in patients with fibromyalgia [20] and rheumatoid arthritis [21]. In Japan, physiological responses during To-ate (remote action), which is a technique to attack an opponent with no physical contact, were investigated in a pair of experts in traditional martial arts and revealed changes in intervals between successive heartbeats (R-R intervals) [22] and alpha phase synchronization [23]. Except for these limited data, no systematic empirical studies have investigated whether and how continued practice of Japanese martial arts may cause desirable changes at the psychological, physiological, or neurocognitive levels.

Thus, in the present study, we conducted a cross-sectional questionnaire survey to examine whether and how continued practice of martial arts as well as expertise in martial arts are associated with higher dispositional mindfulness and psychological health outcomes. A cross-sectional design has an intrinsic limitation in that completely excluding alternative explanations is difficult; for example, those with higher mindfulness and psychological health may tend to continue the practice longer ([10, 12]; see also [2]). In addition, comparisons between practitioners and non-practitioners using a cross-sectional design have a limitation in that any mental attitudes and/or psychological dispositions that are not considered might potentially increase the probability of practicing marital arts. Nevertheless, determining the psychological status in a population of long-term practitioners in which a systematic empirical survey has just started is undoubtedly beneficial, because such data can be utilized as a premise for subsequent longitudinal studies.

Accordingly, a population of continued practitioners of martial arts based on Japanese traditions were included in the present study. Specifically, the practitioners belonged to the specific organizations founded by Hiroyuki Aoki, who is one of the representative experts of martial arts in Japan. Hiroyuki’s thoughts on martial arts are based on the idea of *tenshin*, which refers to an energy that is assumed to fill the whole universe. According to Hiroyuki, the purpose of the practice in martial arts is to integrate oneself with tenshin and the great nature, thereby living in harmony with people and the society [24]. For such purposes, practitioners engage in multiple styles of mind-body practices including swordsmanship, karate, bojutsu (art of using sticks as a weapon), as well as their combinations. These styles of practices are considered to have elements comparable to those of sports in that they involve continued physical training, although these practices do not aim to win the games as typical sports do. Besides founding his own method named *Shintaido* (New Body Way), Hiroyuki also established more modern forms of marital arts such as *Kenbu Tenshin Ryu*. Despite these apparent novelties, all his practices are totally grounded on the Japan’s traditions of marital arts [24]. Given the previous findings obtained from other relevant populations of practitioners in Japan, we expected that more extensive practice and expertise in martial arts would predict higher self-reported mindfulness and more desirable psychological health outcomes such as higher subjective well-being and lower depression.

## Methods

### Participants and procedure

Research design for the present study was generally based on preceding cross-sectional studies involving yoga practitioners [10] and speed-reading trainees [12]. Thirty-three healthy practitioners of martial arts (11 females and 22 males; age 22–69 years; mean age = 44.9 years, standard deviation (*SD*) = 12.9) participated as a practitioner group. All these practitioners were members of either a general incorporated foundation or a non-profit organization, both of which were located in Tokyo, Japan, and were founded by the same originator (Hiroyuki Aoki) to promote multiple styles of martial arts practice based on Japanese traditions. According to the self-reports of these practitioners, 22 held the Dan levels and another six held the Kyu levels within the original grading systems of the organizations. Specific content of their daily practice included swordsmanship, karate, bojutsu, etc. and combinations of these. The practitioners reported that they had practiced martial arts for 0.6–35.0 years (mean = 14.9 years; *SD* = 11.8). The practitioners also self-reported that the mean frequency of the practice of martial arts was 2.0 days per week (*SD* = 1.7), which corresponded to 188.7 min per week on average (*SD* = 152.5). Representatives of the two groups and/or the researcher (Hiromitsu Miyata) asked all members of the two groups to participate in the study, unless these members no longer engaged in practice of martial arts at the time of the survey. Distribution and collection of the questionnaires were carried out either by using questionnaires printed on A4 paper or via an e-mail.

In addition, 66 healthy non-practitioners of martial arts (22 females and 44 males; age, 22–67 years; mean age = 44.5 years, *SD* = 11.6) participated as a control group. Data collection for these participants were conducted after the survey for the practitioners had been completed. On the basis of their self-reports, none of these non-practitioners practiced martial arts nor engaged in other relevant contemplative/mind-body practices including Zen, yoga, etc. According to previous studies [10, 12], data collection for the non-practitioners was performed by using “i Research,” i.e., an online survey system by NEO Marketing Inc., Tokyo, Japan. In this system, there were approximately 6,390,000 monitors in Japan who had agreed to participate in multiple online questionnaire surveys by providing demographic information. Non-practitioners of marital arts were matched with practitioners of martial arts on demographic variables including sex (33% females, 67% males), age range (12% in their twenties, 27% in their thirties, etc.), marital status (44% married, 56% unmarried), annual household income level (18% below 2,000,000 Japanese yen, 36% 2,000,000–4,990,000 Japanese yen, 30% 5,000,000–8,990,000 Japanese yen, 3% above 9,000,000 Japanese yen), and living area (Tokyo and the neighboring prefectures, Gunma, Shizuoka, Ishikawa, or Kyoto prefectures). Regarding household income level, the remaining 12% of the participants for both groups answered that they do not know their household income level. The survey included this option in order not to force all participants to report their income. Question items for the non-practitioners were the same as those for the practitioners. Non-practitioners gave answers to the questions that appeared on an online survey display by checking relevant checkboxes. In the survey for the non-practitioners, answers were required for all the question items, so that there were no items left unanswered.

### Psychological scales

The survey for both groups included the Japanese versions of the psychological scales listed below. All these psychological scales had been developed and validated in the preceding studies, and had also been used in the preceding studies involving yoga practitioners [10] and speed-reading trainees [12]. Participants from both groups were notified that the aim of the study involved assessment of the normal psychological status of each participant and had nothing to do with evaluation or superiority/inferiority of any individual. Also, participants from neither group were notified that the study compared practitioners and non-practitioners of martial arts. In addition, the survey instructed participants to always give plain and honest answers. Another psychological scale on state-trait anxiety was included in the survey, the data for which were not analyzed due to technical errors in the instructions/question items.

#### Five facet mindfulness questionnaire (FFMQ)

The FFMQ [25] is one of the most commonly used psychological scales to measure dispositional mindfulness. The scale involves five core dimensions of mindfulness (facets/subscales), i.e., *observing*, *describing*, *acting with awareness*, *non-judging of inner experience*, and *non-reactivity to inner experience*. Each of the 39 items is rated on a five-point scale from 1 to 5. Sugiura et al. [26] developed and validated a Japanese version of the FFMQ by involving Japanese students, which was used in the present study. The present study expected that measures pertaining to martial arts should predict higher dispositional mindfulness, because martial arts are supposed to have similar correlates to mindfulness and/or Zen as well as sports.

#### Subjective well-being scale (SWBS)

The SWBS [27] is a psychological scale developed and validated in Japanese to measure core components of subjective well-being (see also [28, 29] for former versions of the scale). The SWBS has 15 items, each rated on a four-point scale from 1 to 4. These items are classified into the five subscales each containing three items: *general well-being* – *positive affect*, *confidence in coping*, *expectation-achievement congruence*, *general well-being* – *negative affect*, and *transcendence*. The present study again expected that practice of martial arts should predict higher subjective well-being, based on the preceding studies suggesting that dispositional mindfulness is positively associated with psychological well-being ([30, 31]; see also [10, 12]),

#### Beck depression inventory (BDI)

The BDI [32, 33] is one of the common classical measures of depression. The BDI has 21 items, each concerning core symptoms of depression, which are summed to calculate a total score. Each item has four self-evaluative sentences as options, each being scored from 0 to 3. For each item, participants were instructed to select a sentence that best described how they recently felt. The present study used a validated Japanese version of the BDI [34, 35]. Similar to the above, we expected that practice of martial arts should predict lower depression, because increase in dispositional mindfulness has been suggested to improve psychological health [31, 36].

### Statistical analysis methods

All statistical analyses were conducted by using SPSS Statistics 25.0 software. Data for all participants from the two groups were included in the analysis. The initial analyses involved a description of outcomes from the psychological scales and comparisons between the groups. After calculation of total scores for the FFMQ, SWBS, and BDI as well as subscale (facet) scores for the FFMQ and SWBS for each group, independent samples *t*-tests were used to compare the two groups for all these total/subscale scores. As a measure of internal consistency, Cronbach’s alphas (αs) were examined for each group and total/subscale score. Items that were left blank for these psychological scales accounted for 0.16–3.64% for the practitioners and 0.00% for the non-practitioners. Unavailable data for the practitioners were not included in the analysis. Also, to indicate how scores from each scale are associated with each other, Pearson’s correlation coefficients (*r* values) were calculated between the total scores from the FFMQ, SWBS, and BDI. These correlation analyses were conducted separately for each group.

Next, we examined whether and how practice and/or expertise in martial arts would be correlated with psychological outcomes among the group of practitioners. To indicate practice/expertise in martial arts, the following self-reported measures were considered. (1) The *Dan/Kyu rank* refers to the rank or grade used to indicate each practitioner’s degree or level of expertise within each practice of martial arts. The organizations to which participants in the present study belonged had multiple original Dan/Kyu grading systems (e.g., 5th to 1st Dans and 1st to 10th Kyus from higher to lower ranks in bojutsu), and multiple practitioners self-reported that they held two or more different Dans and/or Kyus at the time of the survey. Thus, in the present study these practitioners were categorized according to whether s/he held the Dan rank(s) in either of the ranking systems, held only the Kyu rank(s), or held neither of these ranks (2 = Dan holder, 1 = Kyu holder, 0 = non-Dan/Kyu holder). Spearman’s rank correlation coefficients (*r*_*s*_ values) were calculated to indicate zero-order correlations between the Dan/Kyu rank and total/subscale scores from the psychological scales. (2) The *practice period* indicates the length of time (months) elapsed since the practitioner started to practice marital arts. (3) The *practice frequency* is the number of days in which each participant engaged in the practice of martial arts per week. Finally, (4) the *practice time* points to the total length of practice of marital arts in minutes per week for each practitioner. Regarding the latter three measures, Pearson’s correlation coefficients were examined among the practitioners to look for correlations between practice in martial arts and psychological outcomes. When reporting practice frequency and practice time, four practitioners noted that meditation was included in the specific content of daily practice (frequency or time for meditation were not reported separately). The remaining content of practice for all the practitioners, i.e., swordsmanship, karate, bojutsu, etc., all involved body movements as in sports. To analyze elements of practice comparable to sports, values for these two measures from these four practitioners were excluded from analysis. Distributions for these three measures were positively skewed (skewness = 0.36 for practice period, 1.48 for practice frequency, and 1.31 for practice time), showing that numbers of relatively extensive practitioners were small. Thus, data for these measures were log transformed (base 10) for this and subsequent statistical analyses to reduce the skewness.

Because multiple measures pertaining to martial arts showed statistically significant correlations with the total/subscale scores from the psychological scales, multiple regression analyses using a stepwise method were further conducted with each total/subscale score as a dependent variable. These analyses again involved the group of practitioners, and were conducted for scales/subscales in which at least one measure pertaining to martial arts showed a statistically significant correlation with the scores. These analyses primarily intended to consider the potential influence of demographic variables on the scores from the psychological scales. In addition to the abovementioned four measures relevant to martial arts, we entered the following demographic variables as independent variables in a stepwise manner: sex (0 = male, 1 = female), age (years), marital status (0 = married, 1 = unmarried), and annual household income level (1 = below 2,000,000 Japanese yen, 2 = 2,000,000–4,990,000 Japanese yen, 3 = 5,000,000–8,990,000 Japanese yen, 4 = above 9,000,000 Japanese yen).

## Results

### Reliability, descriptive statistics, and group comparisons

Cronbach’s alpha values for both groups of participants showed overall good to acceptable reliability for the total/subscale scores from the psychological scales, even though alpha values were low for some subscale scores (e.g., α = 0.48 for the *expectation-achievement congruence* subscale scores from the SWBS for the practitioners). Table 1 summarizes mean total/subscale scores from the psychological scales for each group of participants and comparisons between groups. For the FFMQ, the total and two subscale scores, i.e., *observing* and *non-reactivity*, were significantly higher for the practitioners than for the non-practitioners. We found no statistically significant difference between groups for the remaining three subscales. Regarding the SWBS, the total and all five subscale scores were significantly higher for the practitioners than for the non-practitioners. In contrast, total scores of the BDI were significantly lower for the practitioners than for the non-practitioners. Thus, practitioners of martial arts self-reported significantly higher mindfulness and subjective well-being and lower depression compared with the non-practitioners.

**Table 1:** Descriptive statistics and comparisons between groups for each psychological scale

	Practitioners (*N* = 33)		Non-Practitioners (*N* = 66)		Comparisons: Practitioners vs. Non-Practitioners	
	α	Mean (*SD*)	α	Mean (*SD*)	*t* (*p*)	Cohen’s *d*
FFMQ Total	0.91	130.39 (17.97)	0.78	119.14 (12.99)	3.078 (0.004**)	0.76
Observing	0.76	27.61 (4.88)	0.87	20.70 (5.85)	5.787 (< 0.001***)	1.25
Describing	0.78	23.94 (4.59)	0.73	23.83 (4.76)	0.105 (0.917)	0.02
Acting with Awareness	0.71	27.48 (4.30)	0.89	28.52 (6.04)	−0.950 (0.345)	0.19
Non-Judging	0.88	27.94 (6.35)	0.92	26.85 (6.86)	0.757 (0.451)	0.16
Non-Reactivity	0.84	23.61 (4.98)	0.85	19.24 (5.32)	3.888 (< 0.001***)	0.84
SWBS Total	0.90	45.97 (7.30)	0.93	35.65 (9.29)	5.317 (< 0.001***)	1.19
General Well-Being: Positive Affect	0.83	10.43 (1.50)	0.87	7.62 (2.32)	7.024 (< 0.001***)	1.35
Confidence in Coping	0.90	9.06 (2.24)	0.88	7.97 (2.24)	2.246 (0.027*)	0.49
Expectation-Achievement Congruence	0.48	8.42 (1.34)	0.82	6.79 (2.40)	4.233 (< 0.001***)	0.77
General Well-Being: Negative Affect^a^	0.79	9.44 (2.18)	0.82	6.64 (2.23)	5.809 (< 0.001***)	1.26
Transcendence	0.77	8.81 (1.93)	0.74	6.64 (2.06)	4.948 (< 0.001***)	1.08
BDI Total	0.91	9.25 (9.16)	0.92	13.79 (11.02)	−1.996 (0.049*)	0.43

Mean total/subscale scores and their standard deviations (*SDs*) are shown for each scale. *: *p* < 0.05; **: *p* < 0.01; ***: *p* < 0.001

^a^: Higher scores show that less negative affect was self-reported

### Correlations between scales

For the practitioners of martial arts, total scores of the FFMQ were significantly and positively correlated with total scores of the SWBS (*r* = 0.733, *p* < 0.001), and negatively with total scores of the BDI (*r* = − 0.626, *p* < 0.001). Total scores of the SWBS and the BDI showed a significant negative correlation as well (*r* = − 0.820, *p* < 0.001). These correlation trends between the total scores were generally similar for the non-practitioners, i.e., between the FFMQ and the SWBS (*r* = 0.540, *p* < 0.001), between the FFMQ and the BDI (*r* = − 0.494, *p* < 0.001), and between the SWBS and the BDI (*r* = − 0.739, *p* < 0.001). These data show that desirable and non-desirable psychological functions are associated with each other, not only for those practicing martial arts but also for those with no daily practice of martial arts or meditation.

### Measures pertaining to martial arts and psychological outcomes

Among the measures pertaining to practice/expertise in martial arts, the Dan/Kyu rank showed significant positive correlations with the practice period (*r*_*s*_ = 0.776, *p* < 0.001), practice frequency (*r*_*s*_ = 0.604, *p* = 0.0011), and practice time (*r*_*s*_ = 0.633, *p* < 0.001). Also, the practice period showed a significant positive correlation with practice frequency (*r* = 0.484, *p* = 0.011), but not with practice time (*r* = 0.367, *p* = 0.066). Practice frequency and practice time were significantly and positively correlated with each other (*r* = 0.663, *p* < 0.001). Thus, multiple parameters of martial arts were significantly associated with each other.

**Fig. 1:**
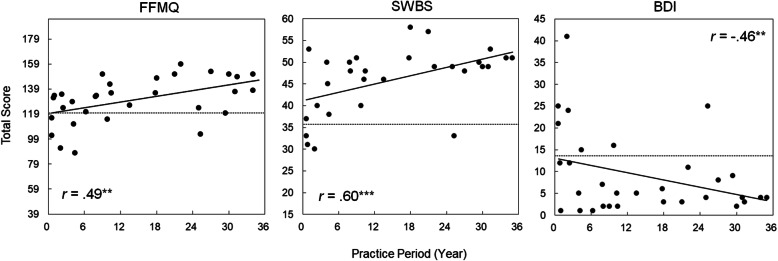
Scatterplots showing correlations between the practice period in years and total scores from each psychological scale, plotted for each practitioner of marital arts. Dotted lines show mean total scores for the non-practitioners. *R*: Pearson’s correlation coefficient. **: *p* < 0.01; ***: *p* < 0.001

For the group of practitioners, Table 2 shows correlation coefficients between measures pertaining to practice/expertise in martial arts and total/subscale scores from the psychological scales. The Dan/Kyu rank showed significant positive correlations with the total scores of the FFMQ and two subscale scores of the SWBS, i.e., *general well-being* – *positive affect* and *general well-being* – *negative affect*, although correlations failed to reach statistical significance for the other total/subscale scores. The practice period was significantly and positively correlated with the total FFMQ scores and two of its subscales, i.e., *observing* and *describing*, positively correlated with the total and all five subscale scores of the SWBS, and negatively correlated with the total score of the BDI (see also Fig. 1 for scatterplots depicted for the total scores). Practice frequency showed significant positive correlations with the total scores of the SWBS and its subscale, i.e., *general well-being* – *negative affect*, and a significant negative correlation with the total score of the BDI; correlations were not statistically significant for the remaining total/subscale scores. In contrast, practice time failed to show statistically significant correlations with any of the total/subscale scores from the psychological scales. These data show that certified ranks showing expertise in martial arts, as well as the length of the practice period and (to a lesser extent) frequency of daily practice, were significantly associated with dimensions of mindfulness and psychological health among practitioners of martial arts.

**Table 2:** Zero-order correlations between measures of practice/expertise and total/subscale scores from the psychological scales among practitioners of martial arts

	Dan/Kyu Rank	Practice Period (months)	Practice Frequency (day per week)	Practice Time (min per week)
FFMQ Total	.398*	.493**	.123	−.027
Observing	.199	.383*	−.094	−.094
Describing	.212	.438*	.142	−.114
Acting with Awareness	.269	.288	.023	.168
Non-Judging	.292	.324	.230	−.024
Non-Reactivity	.286	.279	.131	−.014
SWBS Total	.331	.602***	.435*	.306
General Well-Being: Positive Affect	.521**	.578**	.367	.267
Confidence in Coping	.162	.375*	.264	.096
Expectation-Achievement Congruence	.296	.449*	.168	.129
General Well-Being: Negative Affect^a^	.468**	.522**	.436*	.056
Transcendence	.339	.550**	.280	.263
BDI Total	−.225	−.463**	−.504**	−.352

For the Dan/Kyu rank, Spearman’s rank correlation coefficients (*r*_*s*_ values) are shown for each comparison. For the other three measures of practice, Pearson’s correlation coefficients (*r* values) are shown. *: *p* < 0.05; **: *p* < 0.01; ***: *p* < 0.001

^a^: Higher scores show that less negative affect was self-reported

Results obtained from further multiple regression analyses are summarized in Tables 3 and 4. Regarding the total scores from each scale as shown in Table 3, a longer practice period significantly predicted higher scores of the FFMQ (*R*^*2*^ = 0.272, *B* = 20.118, *SE* = 7.983, *β* = 0.522, *t* = 2.520, *p* = 0.022). Also, a higher practice frequency significantly predicted lower total scores of the BDI (*R*^*2*^ = 0.213, *B* = − 14.044, *SE* = 6.542, *β* = − 0.462, *t* = − 2.147, *p* = 0.047). Regarding the subscale scores as shown in Table 4, a longer practice period significantly predicted higher scores for the *describing* subscale from the FFMQ (*R*^*2*^ = 0.443, *B* = 5.672, *SE* = 1.541, *β* = 0.666, *t* = 3.681, *p* = 0.002). Also, a higher practice frequency significantly predicted higher scores on the *general well-being* – *negative affect* subscale from the SWBS (*R*^*2*^ = 0.246, *B* = 2.926, *SE* = 1.243, *β* = 0.496, *t* = 2.355, *p* = 0.031). Marital status, though not the remaining independent variables, significantly predicted scores on the *transcendence* subscale from the SWBS (scores were higher for those being married; *R*^*2*^ = 0.217, *Β* = − 1.833, *SE* = 0.845, *β* = − 0.466, *t* = − 2.170, *p* = 0.045). All other measures pertaining to martial arts and demographic variables were removed from these regression models (Tables 3 and 4 show beta coefficients when these variables were entered into the models). Variance Inflation Factor (VIF; a measure of multi-collinearity) proved to be 2.954 or smaller for all these regression models, indicating low multi-collinearity. With regard to the remaining subscales, no independent variables significantly predicted psychological outcomes. These data show that longer practice or higher frequency of daily practice of martial arts was significantly associated with higher dispositional mindfulness and dimensions of psychological health, even when multiple demographic variables were taken into account.

**Table 3:** Results of the multiple regression analyses conducted for the total scores from each psychological scale

Independent variable	FFMQ			BDI		
	*β*	*t*	VIF	*β*	*t*	VIF
(Constant)	–	5.063***	–	–	4.726***	–
Demographic variables						
Sex	.146	.667	1.081	−.130	−.508	1.352
Age	.148	.658	1.144	−.206	−.947	1.016
Marital status	−.060	−.221	1.601	.179	.751	1.199
Household income	.152	.722	1.000	−.365	−1.806	1.000
Practice/expertise in martial arts						
Dan/Kyu rank	−.399	−1.291	2.320	.042	.168	1.271
Practice period	.522	2.520*	1.000	−.264	−1.096	1.268
Practice frequency	−.005	−.022	1.268	−.462	−2.147*	1.000
Practice time	−.122	−.538	1.154	.166	.437	2.954
*R*^*2*^	.272			.213		
Adjusted *R*^*2*^	.229			.167		
*F*	6.351*			4.609*		

Variables removed from each model are shown in the table. *: *p* < 0.05; ***: *p* < 0.001

**Table 4:** Results of the multiple regression analyses conducted for the subscale scores from each psychological scale

Independent variable	FFMQ: Describing			SWBS: General Well-Being–Negative Affect			SWBS: Transcendence		
	*β*	*t*	VIF	*β*	*t*	VIF	*β*	*t*	VIF
(Constant)	–	3.360**	–	–	20.415***	–	–	8.231***	–
Demographic variables									
Sex	.303	1.695	1.081	.123	.491	1.352	.235	1.075	1.046
Age	.131	.667	1.144	.201	.944	1.016	−.061	−.262	1.106
Marital status	−.147	−.632	1.601	−.155	−.663	1.199	−.466	−2.170*	1.000
Household income	−.049	−.265	1.000	.275	1.334	1.000	−.106	−.481	1.000
Practice/expertise in martial arts									
Dan/Kyu rank	−.309	−1.130	2.320	.124	.511	1.271	−.246	−1.068	1.158
Practice period	.666	3.681**	1.000	.282	1.205	1.268	.016	.057	1.601
Practice frequency	−.114	−.548	1.268	.496	2.355*	1.000	.259	1.110	1.199
Practice time	−.036	−.180	1.154	−.278	−.757	2.954	.335	1.543	1.108
*R*^*2*^	.443			.246			.217		
Adjusted *R*^*2*^	.411			.202			.171		
*F*	13.547**			5.544*			4.707*		

Variables removed from each model are shown in the table. *: *p* < 0.05; **: *p* < 0.01; ***: *p* < 0.001

## Discussion

Compared with non-practitioners, practitioners of martial arts self-reported answers that were calculated as significantly higher scores on mindfulness and subjective well-being and lower scores on depression. Among practitioners of martial arts, the practice period and (to a lesser extent) certified ranks of expertise (Dan/Kyu rank) as well as the frequency of daily practice were significantly correlated with higher mindfulness and psychological health outcomes. These associations between practice/expertise in martial arts and psychological outcomes were generally consistent after controlling for demographic variables, whereas a higher frequency of daily practice, but not a longer practice period, predicted lower depression. These data are overall consistent with the hypotheses and support the notion that continued practice as well as the degree of expertise are associated with higher dispositional mindfulness and desirable psychological status among a population of practitioners of Japanese marital arts.

Results obtained from the present study seem to be consistent with the nature of martial arts. That is, Japanese traditional martial arts are considered not only to involve elements of sports including physical exercise, but also to have close connections with Zen Buddhism [16, 17]. For example, martial arts stresses the importance of both interoceptive and exteroceptive awareness. Such characteristics also seem to overlap with essential components of mindfulness [25, 26]. Also, the present data are generally consistent with those previously obtained for other populations of practitioners of mind-body or contemplative practices in Japan. That is, both yoga practitioners [10] and trainees of meditation-based speed-reading [12] self-reported higher mindfulness and psychological health associated with the period of practice and/or attained stages of expertise. Considering the present data together with those from preceding studies, we could suggest that practice of multiple Eastern mind-body practices might be associated with similar psychological outcomes including higher mindfulness and psychological health. The results also seem important in that they support the view that mind-body practices based on traditions are associated with higher trait mindfulness and psychological health even with no explicit emphasis on mindfulness as in the MBSR [6, 7] or MBCT [8] programs.

Despite these novelties and perspectives, the present study does have important limitations. The first concerns the cross-sectional design of the study. With the present data alone, there could be a counterargument that long-term practitioners had exhibited higher mindfulness and psychological health since before they started the practice of martial arts. Also, the practitioners and non-practitioners in the present study were matched on observable demographic variables alone. It could potentially be possible that differences in mental attitudes and/or psychological characteristics that were not explicitly observed (e.g., general motivational states before starting practice) may have heightened the probability of practicing martial arts among the population of practitioners. These issues are difficult to be resolved by using a cross-sectional design. To overcome these limitations, one potential next step would be to conduct a longitudinal study to follow each practitioner for at least several months or years. For example, including university students who practice marital arts as an extracurricular activity is feasible, because this approach would enable systematic tracing of each practitioner for at least several years. In addition, it should be ideal if we could randomly assign non-practitioners to the practice group and the control group, and follow the same populations for years or even decades by using a longitudinal design. However, these long-term longitudinal studies are impractical, if not impossible to conduct. In this sense, a cross-sectional design also seems to make a significant sense. As indicated in preceding studies [2, 10, 12], a cross-sectional design enables involvement of large numbers of long-term practitioners and highly advanced experts to uncover their psychological, somatic physiological, and/or neurocognitive processes. As noted in the Introduction, use of this design is also useful when first studying a certain population of practitioners.

A second and related issue is that we failed to study sufficient numbers of practitioners to conduct structural equation modeling analyses (e.g., [12]). This may explain why causal relationships between the practice of martial arts and mindfulness and psychological health were not determined more convincingly. Because collecting data from larger numbers of practitioners from the studied populations was practically difficult, involving multiple populations of practitioners who practice different styles of martial arts would be more preferable. Such a widened survey should enable assessment of both similar and different aspects of psychological status between multiple populations of practitioners, and contribute to heightened degrees of external validity. These approaches seem particularly interesting considering the fact that traditional martial arts in Japan have many schools with both commonalities and differences in their practice styles [16].

## Conclusions

Taken together, to the best of our knowledge, the present study is the first to demonstrate dispositional mindfulness and psychological health in a population of continued practitioners of martial arts based on Japanese traditions. Similarity of the present results to those previously obtained in other Japanese practitioners of mind-body or contemplative practices would support the notion that continued practice of multiple Eastern mind-body practices may be associated with higher dispositional mindfulness and psychological health outcomes. Based on these findings, future studies may examine various dimensions of psychological, behavioral, and physiological correlates of martial arts. Comparable to mindfulness training [6–8], martial arts essentially emphasize the importance of attention to one’s own breath and body, and awareness of both the internal and external environments. Introduction of a task to assess interoceptive awareness such as a heartbeat detection task [37] and a water load test [38] combined with behavioral tasks of attention will be promising to examine how performance on these tasks are associated with expertise in marital arts. Because martial arts involve quick, balanced, and flexible body movements as well as mindfulness [16, 18], investigating how mindfulness and psychological statuses may be coupled with capabilities of body balance and coordination among the practitioners will also be challenging [19]. Because many of these components of martial arts seem to overlap with those of sports, making direct comparisons between martial arts and sports by using the same research paradigms should be an important frontier as well.

From a theoretical perspective, Eastern thoughts on mind and body have assumed that mind-body integration and unity should be sought for through physical/mental practices, as termed Shin-Shin Ichinyo (body and mind as one) [3]. This seems to also go in line with Ken-Zen Ichinyo (oneness of Zen and swordsmanship) as has been worded in the traditions of martial arts [17]. Such traditional words seem to represent Japanese forms of mindfulness, and could potentially be well understood within the modern scientific framework of mindfulness. Abovementioned various empirical studies should promisingly contribute to this exciting literature, and lead to an integrated understanding of the traditional mind-body theory in the East and the modern spirituality developed in Western countries.

## Data Availability

The datasets used and/or analyzed during the study are available from the corresponding author upon reasonable request.

## Abbreviations

BDI: Beck Depression Inventory
FFMQ: Five Facet Mindfulness Questionnaire
MBCT: Mindfulness-Based Cognitive Therapy
MBSR: Mindfulness-Based Stress Reduction
SWBS: Subjective Well-Being Scale
